# Interventions to Improve Timely Detection and Diagnosis of Cancer Among the Adult Population: A Scoping Review

**DOI:** 10.3390/healthcare14121600

**Published:** 2026-06-06

**Authors:** Mariam Safi, Bettina Ravnborg Thude, Emma Weiss Christensen, Dorte Ejg Jarbøl, Neringa Andziule, Heidi Lynge Søfelt, Donna Lykke Wolff, Linda Juel Ahrenfeldt, Frans Brandt

**Affiliations:** 1 Internal Medicine Research Unit, University Hospital of Southern Denmark, 6200 Aabenraa, Denmark; brt@rsyd.dk (B.R.T.); emld@rsyd.dk (E.W.C.); neringa.andziuliene@hotmail.com (N.A.); heidi.lynge.sofelt@rsyd.dk (H.L.S.); donna.wolff@rsyd.dk (D.L.W.); fbk@rsyd.dk (F.B.); 2 Department of Regional Health Research, University of Southern Denmark, 5230 Odense, Denmark; 3 Research Unit for General Practice, Department of Public Health, University of Southern Denmark, 5000 Odense, Denmark; djarbol@health.sdu.dk (D.E.J.); lahrenfeldt@health.sdu.dk (L.J.A.)

**Keywords:** intervention, improvements, cancer, early detection

## Abstract

**Highlights:**

**What are the main findings?**

- Three intervention categories were identified to support earlier diagnosis of lung, pancreatic, ovarian, and stomach cancer: public awareness campaigns, education/training initiatives, and direct/streamlined access to diagnostic services.
- Evidence was limited and heterogeneous, but several interventions showed improvements in intermediate outcomes such as timelier investigations, faster diagnostic pathways, and more appropriate referral processes.

**What are the implications of the main findings?**

- Future interventions should combine patient-facing components (awareness) with provider-focused support (training) and system-level access/coordination to reduce diagnostic delays.
- More robust and longer-term evaluations are needed to determine effectiveness across settings and to assess downstream outcomes (e.g., stage at diagnosis, survival, patient-reported outcomes, and cost-effectiveness).

**Abstract:**

Background: This scoping review aimed to identify and evaluate interventions to improve timely detection and diagnosis of lung, ovarian, stomach, and pancreatic cancers among adults. Understanding the effectiveness of these interventions is important for improving cancer care and patient outcomes. Method: A scoping review was conducted following the JBI framework and the PRISMA-ScR Checklist to identify empirical studies reporting interventions focused on lung, stomach, ovarian, and pancreatic cancers. A systematic literature search was performed in three databases (CINAHL, Embase, and Medline). Supplementary searches included citation tracking. English-language empirical studies examining interventions for early cancer detection in adults were included. Open Science Framework was used to register the scoping review protocol (accessed on 11 July 2024). Results: Of 13,142 retrieved citations, 12 articles met the inclusion criteria. Studies were conducted in the UK (*n* = 6), Australia (*n* = 2), Northern Ireland (*n* = 1), Denmark (*n* = 1), and the USA (*n* = 2). Interventions included awareness campaigns, educational programmes, and improved access to health services. Interactive formats and clear messaging increased awareness and help-seeking behaviour. Integrated approaches combining public awareness campaigns with general practitioner educational programmes were associated with promoting timely diagnosis and improving survival rates. Tailored interventions targeting populations such as rural residents and long-term smokers enhanced effectiveness. Conclusion: The review highlights the importance of integrated strategies to improve early cancer detection and diagnosis. Three intervention types were identified: awareness campaigns, educational programmes, and direct access to health services. Future interventions should consider longer campaign durations and tailored approaches for demographic groups, particularly populations in deprived areas, to improve effectiveness.

## 1. Introduction

Cancer is a leading cause of death globally, with approximately 10 million deaths being attributable to cancer and close to 20 million cases of cancer globally in 2022 [1]. In Denmark, cancer also remains the leading cause of death, with around 15,000 deaths and approximately 40,000 new cases diagnosed annually [2] in a population of 5.9 million people [3]. Timely detection and diagnosis are crucial for improving the prognosis of cancer patients, as they enable more effective and less aggressive treatments, thereby enhancing survival rates [4,5,6].

To ensure timely examination, different strategies have been developed in several European countries, including Denmark [7,8]. In 2008, Denmark introduced a standardised cancer patient pathway or fast tracks to provide rapid diagnosis of suspected cancer cases, ensuring uniformity and high-quality care while minimising unnecessary delays in diagnosis and treatment [7]. Currently, most cancer cases in Denmark are detected through these pathways; however, approximately one in four cancer patients are diagnosed outside of established pathways [4]. A recent Danish study [4] found that up to 16% of cancer patients had an unplanned emergency admission within 30 days before their diagnosis, outside the established diagnostic pathways. The one-year mortality rate for these patients was notably high, varying from 18.1% in patients aged 18–39 to 77% in those aged 90 or older [4].

There are multifaceted factors affecting the timely diagnosis of cancer. Previous studies have highlighted the importance of contacting a general practitioner (GP) when experiencing symptoms, as the GPs act as gatekeepers to both out- and inpatient hospital services in many countries [9,10]. Healthcare seeking is a composite process, and several factors, such as physical, social, organisational, and psychological aspects contribute to the decision of whether to seek help or not [11,12]. Whitaker et al. [13] noted that lower health literacy and symptom awareness impact patients’ ability to navigate healthcare systems, while Sætre et al. [14] found that concerns about what the doctor might find were significant barriers to healthcare seeking for lung cancer symptoms.

To address these challenges, it is crucial to explore interventions that enhance timely cancer detection and diagnosis outside of traditional screening and surveillance programmes. We use “timely detection and diagnosis” as an umbrella term for interventions that may promote earlier patient presentation, faster help seeking, improved primary care referral and reduced diagnostic interval at a stage where curative treatment remains possible. This differs from screening, which generally refers to testing asymptomatic people in defined population programmes, whereas this review focuses on symptomatic adults.

This scoping review focuses on four cancers: lung, stomach, ovarian, and pancreatic cancers, which are framed in the literature as ‘hard-to-diagnose’ cancers [15]. These cancers were selected because of their high mortality rates [16,17,18,19], non-specific symptom presentation, diagnostic complexity [15,20] and frequent routes to diagnosis through unplanned admissions [4].

This scoping review aims to identify and map the interventions used to improve timely detection and diagnosis of lung, stomach, ovarian and pancreatic cancers among adults, outside of standard screening programmes and cancer patient pathways. The review will focus on understanding the target populations, characteristics of these populations, outcomes of the interventions, success factors, and recommendations for future interventions.

## 2. Methods

A scoping review was performed following the Joanna Briggs Institute’s (JBI’s) guidance for the conduct of scoping reviews [21]. This framework involves several key components: establishing and aligning the objectives and questions for the review, creating inclusion criteria that correspond with these objectives and questions, and outlining the planned strategy for evidence searching. Additionally, it includes the processes of selecting, extracting, and organising the evidence, summarising the findings in relation to the review’s objectives and questions, and consulting with information scientists, librarians, and other experts throughout the entire process. A protocol for the scoping review was published in the Open Science Framework (OSF) (Registration: https://osf.io/7jtf3/) (accessed on 11 July 2024). The reporting of the review is guided by the PRISMA-ScR Checklist [22] (Supplement S1. PRISMA-ScR Checklist).

### 2.1. Search Strategy

A comprehensive search strategy was developed in consultation with a research librarian and subsequently reviewed by a second librarian specialist. The search terms used were broad, incorporating synonyms, truncations, and combinations of keywords such as ‘early cancer diagnosis,’ ‘delayed diagnosis,’ ‘interventions,’ ‘strategies,’ and ‘patient outcomes’ to ensure all relevant studies that met the population (P), intervention (I), and outcome (O) criteria were captured.

Three scholarly databases, CINAHL, Embase, and Medline, were searched for peer-reviewed articles from January 2014 to 9th of February 2026. These databases were selected in consultation with the research librarian because they provide strong coverage of the biomedical, nursing, allied health, primary care, and healthcare intervention literature relevant to cancer diagnosis and care delivery. These databases were selected because the review focused on healthcare-based interventions to improve timely cancer diagnosis. The complete database-specific search strategies for all three databases are presented in Supplement S2. The decision to select this timeframe was based on our interest in recent and modern interventions. Supplementary searching consisted of backward citation tracking of all included articles. This process identified no additional eligible records. The grey literature was excluded because the review was designed to map peer-reviewed empirical evidence and because a comprehensive grey-literature search across public health and service-level reports was beyond the scope of the project.

### 2.2. Eligibility Criteria

We included empirical studies evaluating interventions aimed at improving timely detection and diagnosis among adults (aged ≥18 years) with one or more of the four targeted cancer types: lung, ovarian, stomach and pancreatic cancer. Eligible intervention could focus on patient experience, clinical outcomes, system performance, or staff experience.

We excluded non-empirical studies (e.g., reviews, perspectives, the grey literature), studies without an intervention, papers that did not include any of the cancer types of interest, papers not published in English, screening studies and studies focused on palliative care. For studies including multiple cancer types, the study was eligible if at least one of the four target cancers was included. The restriction to high-income countries was applied because the review was intended to inform intervention development in health systems broadly comparable to Denmark.

### 2.3. Screening and Data Extraction

All retrieved citations from the literature search were imported into EndNote X9 (Clarivate, London, UK) and Covidence systematic review software (Veritas Health Innovation, Melbourne, Australia; web-based platform, no version number assigned). Following the removal of duplicates, 13,142 records remained for screening. To ensure consistency in applying the eligibility criteria, 100 abstracts were pilot screened independently by reviewers working in pairs (MS, BRT, EWC, DWL, NA, HLS). The remaining titles and abstracts were then screened independently by two reviewers. Disagreements were resolved through discussion or by involving a third reviewer (FB).

**Figure 1:**
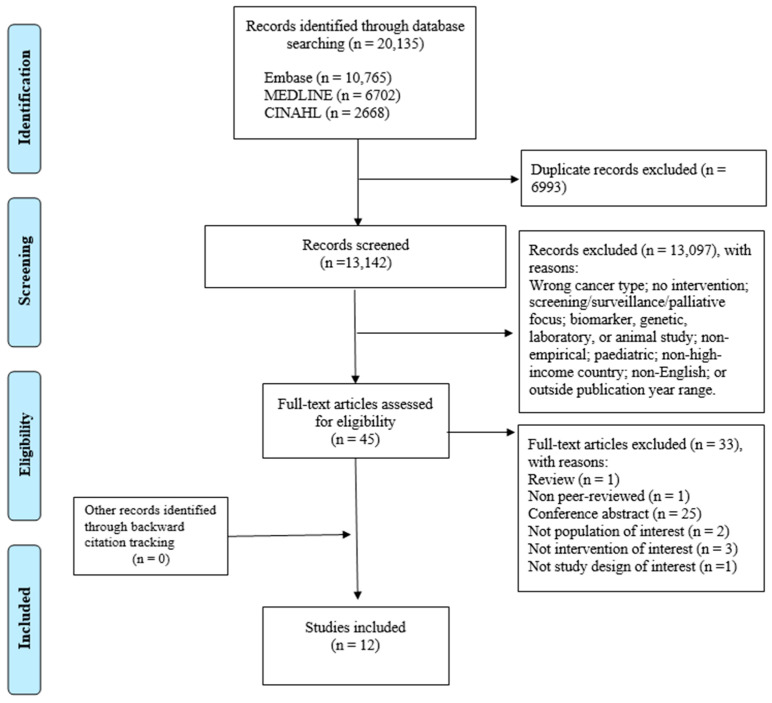
PRISMA flow diagram.

After title and abstract screening, 45 articles were assessed for eligibility. The inclusion criteria were applied independently during the full-text review by three pairs of reviewers (MS, BRT, EWC, DWL, NA, HLS). Disagreements were resolved through discussion or by involving a third reviewer (FB) (Figure 1).

Data related to study characteristics, population, cancer type, country and setting, intervention components, comparator (where applicable), outcome measures (e.g., patient and system), key findings, reported supporting factors, and authors’ recommendations were extracted into a specifically designed spreadsheet. The extraction form was pilot tested using a sample of three included studies and refined before full data extraction. Data extraction was performed independently by two reviewers working in pairs (MS, BRT, EWC, DEJ, LJA, FB). After extraction was completed, one reviewer (MS) cross-checked the extracted data with each reviewer pair for consistency and completeness. Any disagreements were resolved through joint discussion among the reviewers. For multi-cancer studies, we sought to extract data only for the four target cancers. When cancer-specific outcomes were not available, we reported aggregate results only if at least one target cancer was included. These aggregate findings were interpreted cautiously.

### 2.4. Data Mapping and Synthesis

A narrative synthesis [23] was performed for this review. It was guided by the review’s overarching aim and sub-questions. Data synthesis was undertaken in the following stages: (i) evidence mapping (ii) tabular and graphical presentation of the included studies. We organised the dataset by study characteristics, details of the intervention, and reported outcomes. In this scoping review, we assessed intervention effectiveness as reported by the primary study authors in their findings and conclusions. Interventions were considered effective when the primary study authors reported a positive effect. In line with the JBI’s guidance for the conduct of scoping review, this type of review does not include a quality assessment of the included articles. Therefore, findings are presented as reported associations or author-reported effects; definitive conclusions on effectiveness cannot be drawn.

## 3. Results

After removal of duplicates, 13,142 records were screened, and 45 full-text articles were assessed for eligibility. In total, 12 unique published articles met the inclusion criteria. The article selection process is presented in the PRISMA flow diagram (Figure 1).

Table 1 provides an overview of the characteristics of the included studies. The geographic distribution of the published articles included studies from the UK (*n* = 6), Australia (*n* = 2), Northern Ireland (*n* = 1), Denmark (*n* = 1), and the USA (*n* = 2). The studies spanned different countries with a concentration of studies from the UK. These studies used a variety of study designs, including randomised controlled trials, prospective cohort studies, time-trend studies, observational studies, and quasi-experimental designs.

### 3.1. Study Participant Characteristics

The study participants varied widely across the included studies, encompassing both the general population and healthcare professionals. The general population included long-term smokers, older adults, rural residents, women at risk of ovarian cancer, patients with cancer symptoms, patients referred to cancer examinations, and people from low socioeconomic status (SES) backgrounds. Several studies specifically targeted people with long-term smoking history, as this group is at increased risk of developing lung cancer [26,29]. Many interventions focused on individuals aged 50 and above, given that the risk of most cancers increases with age [27,35]. Specific campaigns and educational programmes were tailored for rural residents to address the unique challenges they face in accessing healthcare services, as this group often experiences disparities in healthcare access and outcomes [28]. Although most studies did not explicitly focus on low SES populations, the inclusion of rural populations in some studies indirectly addressed this group. Rural populations often face socioeconomic disadvantages, which can impact their access to healthcare services [28,35]. Additionally, Puckett et al. [34] specifically targeted women at risk of ovarian cancer, many of whom were from low SES backgrounds.

In addition to the general population, healthcare professionals, particularly GPs, were also targeted for specialised training. Several interventions included training and educational programmes for GPs to improve their knowledge and skills in early cancer detection and referral processes. These programmes aimed to enhance the capacity of GPs to identify and refer patients at risk of cancer more effectively [28,30,31].

### 3.2. Interventions

The scoping review included studies that investigated various interventions aimed at improving the timely detection and diagnosis of cancer among adults. The studies covered a range of cancer types, including the four target cancer types: pancreatic, lung, stomach/gastric, ovarian cancer, as well as, in some cases, breast, prostate, hepatocellular carcinoma, and colorectal cancers. Most of the studies addressed lung cancer (*n* = 6). The interventions targeted both the general population and healthcare professionals, employing diverse approaches such as awareness campaigns, educational programmes, and direct access to health services. Because several interventions had multiple components, studies could contribute to more than one intervention category. The included studies reported outcomes at different levels. These included awareness outcomes, such as symptom recognition and cancer knowledge; behavioural outcomes, such as help-seeking intentions and GP attendance; diagnostic-process outcomes, such as imaging rates, urgent referrals, waiting times, and diagnostic intervals; and clinical outcomes, such as stage distribution, emergency presentation, treatment rates, and survival. These outcomes represent different levels of evidence and should not be interpreted equivalently.

### 3.3. Awareness Campaigns

Six studies [25,26,31,32,33,35] focused on mass media and public awareness campaigns to enhance early cancer detection. These campaigns primarily targeted lung and abdominal cancers. The studies reported improvements in public awareness and help-seeking behaviours among diverse participant groups. For instance, mass media campaigns were associated with increases in community-ordered chest X-rays (CXRs) and earlier-stage cancer diagnoses in the UK [26,31]. These campaigns were effective in reducing the number of patients diagnosed at late stages and decreasing emergency presentations [31]. One study from Northern Ireland demonstrated that a co-designed serious anatomy game was associated with improved pancreatic cancer awareness and help-seeking intentions, showcasing the potential of interactive and engaging formats in public health interventions [25]. Additionally, Torrance et al. [35] found that a regional public awareness campaign targeting abdominal cancer symptoms was associated with an increase in GP attendances and urgent referrals for suspected abdominal cancers, emphasising the effectiveness of well-targeted public health campaigns.

### 3.4. Educational Programmes

Four studies [28,29,31,34] implemented educational programmes targeting both the general population and healthcare professionals. These programmes were designed to improve awareness, knowledge, and early detection of cancer symptoms. Educational interventions included community-based symptom awareness campaigns, GP educational programmes, and personalised self-help manuals. The studies reported improvements in outcomes such as increased consultations regarding respiratory complaints and improved knowledge of cancer risk factors and symptoms among participants, which included long-term smokers and rural residents. For instance, the CHEST intervention, which included spirometry and a self-help manual, was associated with an increase in respiratory consultations among high-risk individuals [29]. However, some studies indicated that longer durations and consistent implementation were necessary to achieve substantial reductions in diagnostic intervals [28]. Puckett et al. [34] utilised Inside Knowledge campaign materials to educate both women and healthcare providers about ovarian cancer. The educational sessions were associated with increased knowledge of ovarian cancer risk factors, symptoms, and diagnostic methods among participants. Participants also reported increased confidence in discussing ovarian cancer and greater intentions to seek medical care for symptoms. However, this study did not measure diagnostic activity or clinical outcomes. Its findings therefore mainly relate to awareness and behavioural intentions.

### 3.5. Direct Access to Health Services

Three studies [24,27,30] described interventions that created faster, more direct routes into diagnostic assessment and onward management. These included nurse-led one-stop pathways and GP-initiated access to LDCT, and a national coordination model activated by radiological suspicion. Populations included patients presenting with jaundice, long-term smokers eligible for lung investigation, and patients with imaging suspicious for hepatopancreatobiliary malignancy. These interventions were associated with improved access to imaging and specialist review, reduced avoidable delays, and supported more efficient triage and referral processes. For instance, a nurse-led pancreatic pathway provided direct access to diagnostic imaging, and was associated with reduced waiting times and fewer unnecessary admissions [27]. Similarly, GP training combined with direct access to LDCT was associated with improved referral processes and a higher positive predictive value for fast-track lung cancer referrals [30]. In Adair et al. [24] PHCC-PIP implemented a national Cancer Care Team with radiology alerts, rapid specialist imaging review, and centralised staging coordination. This intervention was associated with shorter pancreatic cancer timelines, including a reduction in median time from imaging report to definitive treatment from 54 to 38 days and from imaging report to GP being informed from 7 to 3 days. It also facilitated earlier redirection of patients whose suspicious imaging ultimately reflected non-cancer diagnoses.

### 3.6. Supporting Factors of the Interventions

Table 2 highlights factors that may have contributed to the success of interventions aimed at improving timely cancer detection and diagnosis. Effective engagement, healthcare professional involvement, and tailoring interventions to specific populations were common features across several studies. Effective engagement was an important component, with interventions using interactive or clear formats and consistent messaging showing improvements in awareness and help-seeking behaviours. For example, engagement was a key feature in the serious game developed by Anderson et al. [25], which was associated with improved pancreatic cancer awareness and intentions to seek help. This may be related to its interactive nature and the involvement of experts, patient advocates, and healthcare professionals in its design. Similarly, the mass media campaigns by Ball et al. [26] and Kennedy et al. [31] used clear and actionable messages and were associated with improvements in outcomes such as increased public awareness and early-stage cancer diagnosis. Healthcare professional involvement was another important factor. Providing direct access to diagnostics and comprehensive GP training facilitated early detection and appropriate referrals. Studies by Clark et al. [27] and Guldbrandt et al. [30] highlighted the potential value of giving GPs direct access to diagnostic tools, such as low-dose CT scans, alongside specialised training. These approaches were associated with quicker access to diagnostic imaging, reduced waiting times, and changes in referral patterns.

Tailoring interventions to specific populations, such as rural residents or high-risk groups, seemed to support timely cancer detection and diagnosis. Emery et al. (2017) [28] and (2019) [29] focused on rural populations and long-term smokers, respectively, addressing some of the unique challenges these groups face in accessing healthcare services. These targeted interventions were associated with improvement in awareness, increased consultations, and behavioural outcomes.

## 4. Recommendations for Future Interventions

The included studies also made some recommendations for future interventions. Studies such as those by Ball et al. [26], Koo et al. [32], and McCutchan et al. [33] highlighted the need for continuous public engagement to maintain heightened awareness levels and encourage timely medical consultations.

Providing continuous education and support for healthcare providers on best practices and new diagnostic approaches was also emphasised. Studies by Emery et al. [28] and Puckett et al. [34] highlighted the importance of continued training for healthcare providers to support the implementation of interventions. Developing tailored interventions that consider the specific needs of different demographic groups, particularly in deprived areas, may substantially enhance the relevance and reach of future interventions. Emery et al. [28] and Torrance et al. [35] indicated the potential benefits of tailoring interventions to address population-specific challenges. Additionally, Emery et al. [28], Ball et al. [26], and McCutchan et al. [33] recommended implementing interventions over a longer period to ensure sustained impact and allow better evaluation of outcomes. Similarly, Adair et al. [24] suggested assessing the sustainability of their national coordination model, developing automated radiology alert systems, and exploring how the approach could be adapted for other aggressive or less survivable cancers.

## 5. Discussion

This scoping review identified 12 studies that evaluated interventions aimed at improving timely detection and diagnosis of lung, ovarian, stomach, and pancreatic cancers in adults outside of standard screening programmes and cancer patient pathways. The review revealed three broad intervention categories that may inform future efforts: (i) awareness campaigns, (ii) educational programmes, and (iii) direct access to health services. Several studies reported that interventions using interactive formats and clear messaging were also associated with improved awareness and help-seeking behaviours among the target populations. Integrated approaches, particularly those combining public awareness campaigns with GP educational programmes, were also associated with early diagnoses and improved clinical outcomes in some studies.

An important finding is that the included studies measured outcomes at different levels, from awareness and behavioural outcomes to diagnostic-process outcomes, and clinical outcomes such as stage distribution, and survival. These outcomes should not be interpreted equivalently.

Most of the available evidence was related to lung cancer. In contrast, evidence for ovarian, stomach, and pancreatic cancers was more limited and heterogeneous. Therefore, the conclusions of this review should not be interpreted as applying equally across all four cancer types. The dominance of lung cancer evidence highlights the need for more focused research on interventions to support timely detection and diagnosis of ovarian, stomach, and pancreatic cancers, which are also associated with non-specific symptoms, diagnostic complexity, and poor prognosis [16].

The reviewed studies highlighted several important considerations for future interventions, including the duration required to assess impact and the need to design interventions that account for barriers to healthcare seeking and access. Although few studies directly targeted socioeconomically disadvantaged populations, some interventions addressed groups that may experience access barriers, such as rural residents or populations at increased cancer risk. A study by Austoker et al. [36] found that awareness-raising campaigns often see higher responses from those at lower risk, with limited evidence of long-term impact. This suggests that future campaigns should consider how to reach populations who may be less likely to respond to general public health messages.

A review about barriers to healthcare seeking with symptoms of different cancer types by McCutchan et al. [37] found that individuals with lower SES report more emotional barriers to healthcare seeking, such as being worried or embarrassed. Previous studies also show that younger groups report more practical barriers, such as being too busy or concerned about wasting the doctor’s time [11]. Additionally, Sætre et al. [14] identified that concerns about what the doctor might find, along with being an immigrant or unemployed, were significant barriers to healthcare seeking for lung cancer. In addition to socioeconomic and demographic barriers, long-term mental health morbidity may influence anticipated help seeking and diagnostic testing for cancer-related symptoms, highlighting the need for interventions that account for psychological as well as structural barriers to timely diagnosis [38].

The studies reviewed provided limited information on the inclusion of patients in the design and development of interventions. Other than Anderson et al. [25], none of the other studies explicitly mentioned patient involvement. The development of the intervention followed a co-design process involving pancreatic cancer patients, experts and advocates. This finding is consistent with another review by Okolio et al. [39] which found limited patient involvement in the design phase of healthcare interventions.

There is growing evidence that co-designing interventions with patients can lead to more effective and sustainable outcomes [40,41]. Future intervention studies should explore how patients and communities can be involved in the design phase.

It was challenging to identify a single intervention or a stand-alone approach from this scoping review that could be implemented in our context due to the diverse nature of the interventions studied, each with its own set of complexities and outcomes. The interventions varied in components, settings, scale, intensity, duration, and outcome measures. Hence, assessing the true effectiveness of these interventions was also difficult. Many studies also focused on intermediate outcomes rather than longer-term clinical outcomes. However, several approaches may be adaptable to different healthcare systems and jurisdictions. Local context, healthcare infrastructure, available diagnostic resources, and referral pathways should therefore be considered when designing and implementing future interventions. Recent evidence also highlights the growing role of machine-learning-based cancer risk prediction models as diagnostic-support tools for symptomatic patients, although further validation and implementation research are needed before routine clinical use [42]. Such approaches may complement awareness, education, and diagnostic access interventions by supporting risk stratification and clinical decision making in symptomatic populations.

## 6. Limitations

This research was limited to interventions that targeted lung, ovarian, stomach, and pancreatic cancers to manage the extensive literature base on cancer and maintain a focused review subject. As such, effective interventions targeting other cancer types may have been valuable for our context but were outside the scope of this review. In addition, most of the included evidence related to lung cancer, while fewer studies focused on ovarian, stomach, and pancreatic cancers. This limits the extent to which conclusions can be applied equally across all four cancer types.

This review included only the published peer-reviewed literature, which may have excluded relevant unpublished material, reports, policy documents, and local evaluations. This is important because public awareness campaigns and service-level interventions are often evaluated outside the peer-reviewed literature. Therefore, the exclusion of the grey literature may have introduced publication bias. The search strategy was developed in collaboration with a research librarian and peer-reviewed by a second librarian specialist. The review was limited to CINAHL, Embase, and MEDLINE as the review focused on healthcare-based interventions. Additional multidisciplinary and behavioural science databases, such as Scopus, Web of Science, and PsycINFO, may have identified further relevant studies, particularly studies focusing on behavioural and educational interventions.

Only interventions published in the English language were included in this study. Studies in languages other than English are likely to be valuable in this area and warrant further investigation. In addition, only studies from high-income countries were included to improve comparability with the Danish healthcare context. The findings may therefore have limited transferability to low- and middle-income countries, where healthcare access, health literacy, diagnostic resources, and referral systems may differ substantially. A systematic review [43] on barriers to cancer diagnosis and care in low- and middle-income countries highlighted financial challenges, geographical obstacles, health-system limitations, and low health literacy as common barriers across the cancer care pathway. Furthermore, the transferability of interventions to other contexts, including the Danish healthcare system, requires careful local adaptation and evaluation.

Additionally, only a small number of relevant studies were identified, and they were highly heterogeneous in terms of intervention types, populations, settings, study designs, and outcome measures. This limited comparability across studies and made it difficult to determine which intervention components were most important. Overall, the evidence base remains sparse and underscores the need for further research on strategies to improve timely cancer detection and diagnosis.

Furthermore, and in accordance with JBI’s guidance for scoping reviews, we did not undertake a quality appraisal or risk-of-bias assessment of the included studies. Therefore, definitive conclusions about the effectiveness of the interventions cannot be drawn. The findings should be interpreted as a mapping of available evidence.

Despite these limitations, the search strategy for this review was developed in collaboration with a librarian and peer reviewed. The selected databases were searched using a broad strategy covering the literature from the last 12 years. This approach helped identify a range of peer-reviewed studies addressing interventions for timely detection and diagnosis of hard-to-diagnose cancers.

## 7. Conclusions

This scoping review included 12 studies and mapped interventions that may support timely detection and diagnosis of lung, ovarian, stomach and pancreatic cancers among adults outside of standard screening programmes and cancer patient pathways. The review identified three broad intervention categories: public awareness campaigns, educational programmes, and direct access to health services. The findings suggest that diverse and integrated approaches may support earlier presentation, improved referral processes, increased diagnostic activity, and reduced diagnostic intervals. However, the evidence was limited and heterogeneous, and most studies focused on lung cancer. Therefore, conclusions about effectiveness should be interpreted cautiously, particularly for ovarian, stomach, and pancreatic cancers. Future research should focus on evaluating the long-term impact of awareness campaigns and exploring the effectiveness of interventions in diverse socioeconomic contexts. In addition, future interventions should involve patients and relevant communities in their design and development to ensure that strategies are patient-centred, and responsive to barriers to timely diagnosis.

## Figures and Tables

**Table 1:** Characteristics of the included studies.

Author, Year, Country	Study Type (Study Period)	Study Aim	Cancer Type(s)	Targeted Population (Sample Size)	Intervention Description	Outcome Measure(s)	Key Findings
Adair et al., 2025, Scotland [24]	Quasi-experimental before-and-after quality improvement study (retrospective and prospective audit). Retrospective: October 2021–December 2022 Prospective: December 2022–December 2023.	To centralise oversight of staging pathways for pancreatic cancer and hepatocellular carcinoma across Scotland in order to streamline diagnosis to definitive treatment and improve communication.	Pancreatic cancer and hepatocellular carcinoma	Adults with radiological suspicion of PC or HCC within the national HPB cancer network (NHS Scotland). Pancreatic cancer subgroup used in this review: (Retrospective *n* = 223, Prospective intervention *n* = 169).	Implementation of a national Cancer Care Team (PHCC-PIP) to coordinate staging after radiological suspicion, using radiology alerts, rapid specialist imaging review, pathway navigation, and shared multidisciplinary communication through definitive treatment.	System performance outcomes (Key Performance Indicators): Time from radiological report to GP/CNS notification, time to MDT discussion, time to treatment plan finalisation, Time to definitive treatment.	Significant reduction in diagnostic and treatment intervals. For pancreatic cancer, median time to definitive treatment decreased from 54 to 38 days (*p* = 0.005). All seven KPIs improved significantly in the intervention cohort.
Anderson et al., 2024, Northern Ireland [25]	Quasi-experimental. Before-and-after design. (1 November 2022–1 December 2022).	To evaluate the effectiveness of serious games on the awareness of symptoms of pancreatic cancer and help-seeking intentions within the general population.	Pancreas	General public (*n* = 727).	A game co-designed with experts, patient advocates, and healthcare professionals. The game presents a human anatomy diagram with sections linked to questions about pancreatic cancer.	Patient outcomes: Symptom awareness scores; help-seeking intentions; usability and educational value of the game.	Significant improvement in pancreatic cancer awareness (*p* < 0.001, d = 1.43) and help-seeking intentions (*p* < 0.001, d = 1.10). High usability and educational value reported by participants.
Ball et al., 2022, UK [26]	Prospective pre-post evaluation (2015–2017).	Evaluation of a national mass media campaign to raise public awareness of possible lung cancer symptoms.	Lung	General public aged 50+ (*n* not specified) (Phase 1 questionnaire 897; and phase 2 questionnaire 833).	A national mass media campaign as part of the ‘Be Clear on Cancer’ programme, focusing on symptoms like persistent cough and shortness of breath.	Patient Outcomes: Awareness of lung cancer symptoms; help-seeking intentions; early-stage diagnoses; 1-year survival. System Outcomes: GP attendances; GP referrals; emergency presentations; number of cancer diagnoses; X-rays and CT scans; outpatient attendance; inpatient admissions; major lung resections.	Small favourable changes in 11 of 15 metrics, including a 2.11% (95% CI 1.02–3.20, *p* < 0.001) improvement in early-stage lung cancer diagnoses. These changes did not lead to an increase in GP attendance. Trends indicated gradual changes rather than immediate impacts from the campaign. Increase in urgent referrals for lung cancer, and a reduction in the number of patients diagnosed due to emergency presentations. Increase in X-rays and CT scans, outpatient attendance, and inpatient admissions. The percentage of patients undergoing major resections rose, as did survival rates.
Clark et al. 2018, UK [27]	Prospective cohort study (8 weeks).	To evaluate the effectiveness of a nurse-led, one-stop jaundice service in diagnosing pancreatic cancer early and improving patient outcomes.	Pancreatic	Patients referred under the two-week wait rule for suspected obstructive jaundice (*n* = 54).	Nurse-led, one-stop jaundice service providing biliary drainage and rapid diagnostic imaging (ultrasound, CT, MRI) and potential interventions like ERCP or percutaneous transhepatic cholangiography (PTC) for patients with obstructive jaundice.	Patient Outcomes: Time to diagnosis; patient satisfaction; rates of curative surgery referrals. System Outcomes: Appropriateness of referrals; hospital bed days.	The service provided earlier opportunities for curative surgery, reduced waiting times for outpatient diagnostic imaging from 10–14 days to 1–5 days, avoided unnecessary hospital admissions, and was positively received by patients.
Emery et al., 2017, Australia [28]	Cluster RCT (1 March 2012–31 March 2014).	To evaluate the effect of community-based symptom awareness campaign and GP educational interventions on time to diagnosis in rural cancer patients.	Lung, Breast, Prostate, Colorectal	Rural population (*n* = 1358) and GP practices (*n* = 56).	Community symptom awareness campaign tailored for rural Australians and GP intervention with symptom risk assessment charts and local cancer referral pathways Trial Area A received the community symptom awareness campaign, while Trial Area B acted as the control. Within each area, general practices were randomised to receive the GP education intervention or act as controls, stratified by practice size.	Patient Outcome: Total diagnostic interval (TDI).	No significant differences in median or mean TDI between intervention and control groups. Limited dose or duration of interventions, or the interventions did not have a measurable effect.
Emery et al., 2019, Australia [29]	RCT (29 May 2013–19 November 2015).	To test the effect of a modified CHEST (Community Health Education and Screening Techniques) intervention on help seeking for respiratory symptoms in people at increased risk of lung cancer in primary care, and measure its effect on consultation rates for respiratory symptoms.	Lung	Long-term smokers, aged 55+ (*n* = 551).	The intervention entailed a consultation to discuss and implement a self-help manual, followed by self-monitoring reminders to encourage help seeking for respiratory symptoms.	Patient Outcomes: Respiratory consultation rates; self-efficacy; knowledge of symptoms; symptom appraisal and help-seeking intervals; anxiety; cancer worry; quality of life. System Outcomes: Health service utilisation.	Intervention led to 40% relative increase in respiratory consultations in the intervention group: (adjusted rates (95% CI) intervention 0.57 (0.47 to 0.70), control 0.41 (0.32 to 0.52), relative rate 1.40 (1.08 to 1.82); *p* = 0.0123). There were no significant differences in time to first respiratory consultation, total consultation rates or measures of psychological harm.
Guldbrandt et al., 2014, Denmark [30]	Cohort study nested in a randomised trial (November 2011–June 2013 (19 months)).	To evaluate the implementation of a technological and organisational initiative in the form of a brief GP update and direct access to low-dose CT (LDCT) from general practice for patients with respiratory symptoms and analyse its impact.	Lung	Patients with respiratory symptoms (*n* = 648) GPs (*n* = 266).	Direct access to LDCT from general practice combined with a Continuing Medical Education (CME) meeting on lung cancer diagnosis.	Patient Outcomes: Detection of lung cancer; stage distribution; patient referral rates; CT scan. System Outcomes: referral rates for LDCT; use of fast-track lung cancer pathways; additional diagnostic work-up needed; GP participation in CME.	During the 19-month period, 648 patients were referred to LDCT. A total of 2.3% were diagnosed with lung cancer, with 60% in an early stage. CME participation was associated with a 61% higher CT referral rate and a more than doubled positive predictive value for lung cancer in fast-track referrals compared to the control group.
Kennedy et al., 2018, UK [31]	Time-trend study (2008–2015).	To assess lung cancer outcomes following a coordinated public awareness campaign and primary care educational programme in Leeds, UK.	Lung	Patients with lung cancer symptoms (*n* = 4759).	Four elements: primary care educational package, marketing communications campaign, community health educators, and self-request CXR service.	Patient Outcomes: Stage distribution of lung cancer cases; route to diagnosis; treatment rates; 1-year survival; mortality rates. System Outcomes: Number of community-ordered chest X-rays (CXRs) performed.	Significant increase in 80.8% in annual community-ordered CXRs, 8.8 percentage point increase in early diagnoses (stage I/II) from 26.5% to 35.3%, 9.3% reduction in absolute number of patients diagnosed at late stages (III/IV) from 1254 to 1137. Significant stage shift towards earlier stage lung cancer, reduction in emergency presentations, increase in radical therapy, and improved 1-year survival from 31.8% to 40.3%. (*p* < 0.0001).
Koo et al., 2018, UK [32]	Observational study (February 2015–July 2017).	To evaluate the effect of the National Oesophagogastric Cancer Awareness Campaign on diagnosis rates and its impact on endoscopy service workload in County Durham.	Oesophageal, Gastric	Patients referred for upper gastrointestinal (UGI) endoscopy (*n* = 406).	The National Oesophagogastric Cancer Campaign with key messages about recognising: (1) Heartburn most days for 3 weeks or more could be a sign of cancer. (2) If food is sticking when you swallow, tell your doctor.	Patient Outcomes: Number of cancer diagnoses; cancer stage. System Outcomes: Endoscopy referral rates; waiting times for elective gastroscopies.	The campaign had no significant impact on the diagnosis of oesophagogastric cancers and increased routine waiting times for elective gastroscopies.
McCutchan et al., 2020, UK [33]	Quasi-experimental before-and-after study (July 2016–September 2016).	To evaluate the impact of a four-week mass-media campaign on awareness, presentation behaviour, and lung cancer outcomes.	Lung	Population-representative samples (pre-campaign: *n* = 1001; post-campaign: *n* = 1013).	National mass-media campaign disseminated through TV, online, bus, radio, and posters, targeting adults over 50 years with the message “If you’ve had a cough for three weeks or more, tell your doctor”.	Patient Outcomes: Cough symptom recall/recognition; worry about wasting doctor’s time; GP visits for cough; number of new lung cancer diagnoses; stage of diagnosis. System Outcomes: GP-ordered chest X-rays; Urgent Suspected Cancer (USC) referrals; radiology requests.	Significant increase in cough symptom recall (13.3%) and recognition (4.4%), and decreased worry about wasting doctor’s time (6.4%). GP visits for cough increased by 29%, GP-ordered chest X-rays increased by 23%. No significant change in USC referrals, new diagnoses, or stage distribution of lung cancer.
Puckett et al., 2018, USA [34]	Pre-post intervention design (2014–2015).	To evaluate the impact of the Inside Knowledge campaign materials on ovarian cancer knowledge among women and healthcare providers.	Ovarian	Women aged 18+ (*n* = 499) healthcare providers (*n* = 365).	Educational sessions using Inside Knowledge campaign materials, including print brochures, fact sheets, and survivor stories. Designed to raise awareness about the symptoms and risk factors associated with ovarian cancer.	Patient Outcomes: Knowledge of ovarian cancer risk factors, symptoms, and diagnostic methods; confidence in discussing ovarian cancer; behavioural intentions related to ovarian cancer information.	Increased knowledge among the women and healthcare providers about key ovarian cancer symptoms (e.g., bloating, pelvic or abdominal pain, difficulty eating, and urinary symptoms) and risk factors. Both women and providers reported a significant increase in confidence in their ability to discuss ovarian cancer. Additionally, women reported an increased likelihood of seeking medical advice if experiencing potential symptoms.
Torrance et al., 2021, UK [35]	Regional public awareness campaign evaluation (9 February to 31 March 2017).	To evaluate the impact of the regional ‘Be Clear on Cancer’ (BCoC) campaign on public awareness of key abdominal cancer symptoms in people aged 50+.	Ovarian, Pancreatic, Stomach, Colorectal, Kidney, Oesophageal	Adults aged 50–89 years from East and West Midlands.	Public awareness campaign targeting abdominal cancer symptoms including educational materials, advertisements (e.g., posters, brochures), media campaigns (e.g., TV, radio), community events, and outreach.	Patient Outcomes: Public awareness; GP attendance for abdominal symptoms; cancer diagnosis rates. System Outcomes: Urgent GP referrals for suspected abdominal cancers.	The campaign region saw significantly higher recognition of the BCoC abdominal campaign compared to the control area (35% vs. 24%, *p* < 0.05). Knowledge of ‘bloating’ as a symptom improved significantly (*p* = 0.03). GP attendances for abdominal symptoms increased by 5.8% (*p* = 0.03), and urgent GP referrals for suspected abdominal cancer rose by 7.6%, compared to a non-significant 0.05% change in the control area. Diagnoses of specific abdominal cancers (colorectal, pancreatic(*n* = 102), and stomach (*n* = 17)) were similar to or higher than the median in the campaign area but not in the control area for people aged 50 and over.

**Table 2:** Study success factors and recommendations for future interventions.

Study	Success Factors	Recommendations for Future Interventions
Adair et al., 2025, Scotland [24]	National centralised coordination via a dedicated Cancer Care Team; clear pathway ownership and active case navigation; standardised, measurable KPI-based monitoring of staging timelines; integration within existing healthcare structures (no structural reorganisation required).	Longer follow-up to evaluate impact on survival and clinical outcomes. Inclusion of patient-reported outcome measures (PROMs) and patient experience data. Assessment of impact on tumour stage at treatment initiation. Formal cost-effectiveness analysis. Development of automated national radiology alert systems. Evaluation of sustainability and long-term integration into routine care. Consider adaptation of the model for other aggressive or less survivable cancers.
Anderson et al., 2024, Northern Ireland [25]	Co-designed serious game with input from experts, patient advocates, and healthcare professionals; interactive and engaging format; high usability and educational value.	Incorporate similar serious game approaches for other hard-to-diagnose cancers; increase accessibility and engagement through mobile platforms to reach a wider audience. Conduct long-term follow-up studies to assess sustained impact on behaviour and outcomes. Address potential technological barriers for older populations.
Ball et al., 2022, UK [26]	Comprehensive mass media campaign; clear and consistent messaging; extensive reach through various media channels; increased public awareness and prompt action.	Extend mass media campaigns to include other symptoms and cancer types; improve GP training and support to handle increased referrals. Develop integrated care pathways to manage increased diagnostic and treatment demands. Implement ongoing public awareness initiatives to maintain heightened awareness levels. Consider the campaign’s impact on healthcare resources and manage potential increases in workload.
Clark et al., 2018, UK [27]	Nurse-led, one-stop service providing immediate access to diagnostic imaging and treatment; streamlined care pathway; high patient satisfaction.	Expand nurse-led one-stop services to other regions and cancer types; integrate with primary care practices for broader reach. Evaluate cost-effectiveness to support wider implementation. Enhance training programmes for nurses to ensure high standards of care. Investigate the scalability of the service in different healthcare settings.
Emery et al., 2017, Australia [28]	Tailored community and GP engagement; targeted awareness campaigns for rural settings; use of local cancer referral pathways and symptom risk assessment charts.	Ensure longer duration and consistent implementation of interventions; focus on personalised education for both patients and GPs. Use telemedicine to reach remote populations. Monitor and evaluate the impact of interventions to refine and improve strategies over time. Address potential variations in healthcare infrastructure and access in rural areas.
Emery et al., 2019, Australia [29]	Behavioural intervention with personalised self-help manual; spirometry to identify at-risk individuals; follow-up reminders to encourage help-seeking behaviour.	Enhance follow-up support for patients’ post-intervention; include digital tools to monitor symptom progression. Strengthen community outreach programmes to increase participation. Collaborate with local health services to provide comprehensive support for at-risk populations. Address limitations such as short intervention duration and participant retention.
Guldbrandt et al., 2014, Denmark [30]	Direct access to low-dose CT (LDCT) from general practice; comprehensive GP training on lung cancer diagnosis; increased awareness and referral rates.	Strengthen GP training on using LDCT and fast-track pathways; expand direct access to LDCT in other regions. Implement continuous professional development programmes to keep GPs updated on best practices. Collect and analyse data to continuously improve referral processes and outcomes. Address potential increases in healthcare costs and manage resource allocation effectively.
Kennedy et al., 2018, UK [31]	Coordinated public awareness campaign; primary care educational programme; significant increase in community-ordered CXRs and earlier stage diagnoses.	Replicate awareness and educational campaigns in other regions; ensure sustained funding for long-term impact. Develop partnerships with local health organisations to support campaign efforts. Use data analytics to identify and target high-risk populations effectively. Address limitations such as potential bias in self-reported data and regional differences in healthcare delivery.
Koo et al., 2018, UK [32]	National campaign with clear, actionable messages; increased GP attendances and urgent referrals for suspected abdominal cancers.	Broaden campaign messages to include additional cancer symptoms; enhance GP capacity to manage increased workload. Introduce routine follow-up for patients presenting with symptoms to ensure timely diagnosis. Implement public health campaigns to reduce stigma and encourage prompt medical consultation. Address potential variations in the impact of the campaign across different demographic groups.
McCutchan et al., 2020, UK [33]	Four-week mass-media campaign with clear messages; significant increase in cough symptom recall and recognition; decreased worry about wasting doctor’s time.	Develop targeted interventions for populations less likely to visit GPs; provide continuous education and support for GPs. Use behaviour change theories to design more effective awareness campaigns. Implement feedback mechanisms to continuously improve the intervention based on participant experiences. Address limitations such as short campaign duration and the need for sustained behaviour change.
Puckett et al., 2018, USA [34]	Educational sessions using Inside Knowledge campaign materials; increased knowledge and confidence among participants; focus on both women and healthcare providers.	Utilise multimedia and social media platforms for wider reach; provide ongoing education and resources for healthcare providers. Develop interactive and engaging educational materials. Establish support networks for women at risk to encourage ongoing vigilance and support. Address potential barriers to participation and retention in educational programmes.
Torrance et al., 2021, UK [35]	Regional public awareness campaign targeting abdominal cancer symptoms; significant increase in GP attendances and urgent referrals for suspected abdominal cancers.	Ensure longer duration and wider reach of the campaign; provide additional resources and training for GPs to manage increased workload. Address potential disparities in campaign impact across different demographic groups. Implement follow-up studies to assess long-term effects on diagnosis and treatment outcomes.

## Data Availability

No new data were created or analysed in this study.

## Supplementary Materials

The following supporting information can be downloaded at: https://www.mdpi.com/article/10.3390/healthcare14121600/s1, Supplement S1: PRISMA-ScR Checklist [22]; Supplement S2: Database-specific search strategies for Medline, Embase and CINAHL.
