# Supplementary material for: Interventions to Improve Timely Detection and Diagnosis of Cancer Among the Adult Population: A Scoping Review
Source: Healthcare (Basel). 2026 Jun 6;14(12):1600. doi: 10.3390/healthcare14121600 (PMC13299167; doi:10.3390/healthcare14121600)
Supplement: Supplementary file 1 [file healthcare-14-01600-s001.zip › S2_Supplement_File_Database specific search strategies for Medline Embase and CINAHL-edited.pdf]

#	**Database: OVID Medline** via SDU Library	**Results from 09-02-2026**
1	"Early Detection of Cancer"/	46,900
2	early diagnosis/	32,987
3	Delayed Diagnosis/	9,503
4	("early detection" or "early diagnosis" or "pre-diagnosis" or "prediagnosis" or "early referral*" or "earlier referral*" or "rapid referral*" or "fast referral*" or "fast track referral*" or "fast referral*" or "referral route" or "fast track" or "referral path?" or "referral interval" or "cancer pathway*" or "cancer care pathway*" or "cancer path?" or "timely detection" or "early identification" or "timeliness" or "time-to-treat*" or "patient interval" or "patient care interval" or "primary care interval" or "general practitioner interval" or "GP interval" or "consult* interval" or "diagnostic* interval" or "time to diagnose*" or "diagnose* stage" or "cancer stage*" or "early diagno?s" or "earlier diagno?s").ti,ab.	324,300
5	1 or 2 or 3 or 4	384,370
6	Early Intervention, Educational/	3,788
7	Internet-Based Intervention/	1,947
8	Psychosocial Intervention/	1,653
9	(intervention* or initiativ* or strateg* or campaign* or aware* or programs* or train*).ti,ab.	4,831,400
10	6 or 7 or 8 or 9	4,832,432
11	lung tumor/	0
12	exp lung cancer/	304,860
13	((lung or pulmonary or bronch*) adj2 (cancer* or neopla* or carcino* or malig* or metasta*)).ti,ab.	320,594
14	11 or 12 or 13	421,734
15	5 and 10 and 14	4,437
16	stomach cancer/ or stomach tumor/ or cardia carcinoma/ or stomach adenocarcinoma/ or stomach carcinoid/ or stomach carcinoma/	120,1001
17	((gastr* or gut or stomach*) adj2 (carcin* or cancer* or neoplas* or tumo* or growth* or adenocarcin* or malig*)).ti,ab.	165,077
18	16 or 17	197,781

19	5 and 10 and 18	1,667
20	exp Pancreatic Neoplasms/	101,803
21	(pancreas* adj2 (neoplas* or cancer* or carcin* or tumo* or metasta* or malig*)).ti,ab.	4,840
22	20 or 21	103,589
23	5 and 10 and 22	9,27
24	exp Ovarian Neoplasms/	103,705
25	(ovarian* adj2 (neoplas* or cancer* or carcin* or tumo* or metasta* or malig*)).ti,ab.	106,157
26	24 or 25	138,460
27	5 and 10 and 26	1,344
28	15 or 19 or 23 or 27	8,099
29	limit 28 to yr="2024 -Current"	2,441

#	**Database: OVID Embase** via SDU Library	**Results from 09-02-2026**
1	"Early Detection of Cancer"/	18,910
2	early diagnosis/	160,317
3	Delayed Diagnosis/	25,220
4	("early detection" or "early diagnosis" or "pre-diagnosis" or "prediagnosis" or "early referral*" or "earlier referral*" or "rapid referral*" or "fast referral*" or "fast track referral*" or "fast referral*" or "referral route" or "fast track" or "referral path?" or "referral interval" or "cancer pathway*" or "cancer care pathway*" or "cancer path?" or "timely detection" or "early identification" or "timeliness" or "time-to-treat*" or "patient interval" or "patient care interval" or "primary care interval" or "general practitioner interval" or "GP interval" or "consult* interval" or "diagnostic* interval" or "time to diagnose*" or "diagnose* stage" or "cancer stage*" or "early diagno?s" or "earlier diagno?s").ti,ab.	507,966
5	1 or 2 or 3 or 4	592,332
6	Early Intervention, Educational/	3,720
7	Internet-Based Intervention/	6,045
8	Psychosocial Intervention/	5,624
9	(intervention* or initiativ* or strateg* or campaign* or aware* or programs* or train*).ti,ab.	6,513,458
10	6 or 7 or 8 or 9	6,516,214
11	lung tumor/	936,90
12	exp lung cancer/	599,851
13	((lung or pulmonary or bronch*) adj2 (cancer* or neopla* or carcino* or malig* or metasta*)).ti,ab.	504,163
14	11 or 12 or 13	734,700
15	5 and 10 and 14	7,456
16	stomach cancer/ or stomach tumor/ or cardia carcinoma/ or stomach adenocarcinoma/ or stomach carcinoid/ or stomach carcinoma/	228,886
17	((gastr* or gut or stomach*) adj2 (carcin* or cancer* or neoplas* or tumo* or growth* or adenocarcin* or malig*)).ti,ab.	243,479

18	16 or 17	310,410
19	5 and 10 and 18	2,734
20	exp Pancreatic Neoplasms/	241,399
21	(pancreas* adj2 (neoplas* or cancer* or carcin* or tumo* or metasta* or malig*)).ti,ab.	9,771
22	20 or 21	243,343
23	5 and 10 and 22	2,711
24	exp Ovarian Neoplasms/	225,551
25	(ovarian* adj2 (neoplas* or cancer* or carcin* or tumo* or metasta* or malig*)).ti,ab.	163,206
26	24 or 25	246,603
27	5 and 10 and 26	2,450
28	15 or 19 or 23 or 27	13,805
29	limit 28 to yr="2024 -Current"	3,995

#	**Database: CINAHL with full text (EBSCO)** via SDU Library	**Results from 09-02-2026**
S1	(MH "Diagnosis+/NU/OG/PF/EV/ED/MT")	420,202
S2	(MH "Early Detection of Cancer")	14,436
S3	(MH "Early Diagnosis+")	33,337
S4	early cancer diagnosis	20,977
S5	early cancer diagnosis	20,977
S6	(MH "Early Detection of Cancer")	14,436
S7	(MH "Diagnosis, Delayed/EV/MT/MO/NU/OG/PF/ST/UT/ED")	217
S8	TI ("early detection" or "early diagnosis" or "prediagnosis" or "prediagnosis" or "early referral*" or "earlier referral*" or "rapid referral*" or "fast referral*" or "fast track referral*" or "fast referral* referral route" or "fast track" or "referral path?" or "referral interval" or "cancer pathway*" or "cancer care pathway*" or "cancer path?" or "timely detection" or "early identification" or "timeliness" or "time-to-treat*" or "patient interval" or "patient care interval" or "priamary care interval" or "general practitioner interval" or "GP interval" or "consult* interval" or "diagnostic* interval" or "time to diagnose*" or "diagnose* stage" or "cancer stage* early diagno?s" or "earlier diagno?s")	10,693
S9	AB ("early detection" or "early diagnosis" or "prediagnosis" or "prediagnosis" or "early referral*" or "earlier referral*" or "rapid referral*" or "fast referral*" or "fast track referral*" or "fast referral* referral route" or "fast track" or "referral path?" or "referral interval" or "cancer pathway*" or "cancer care pathway*" or "cancer path?" or "timely detection" or "early identification" or "timeliness" or "time-to-treat*" or "patient interval" or "patient care interval" or "priamary care interval" or "general practitioner interval" or "GP interval" or "consult* interval" or "diagnostic* interval" or "time to diagnose*" or "diagnose* stage" or "cancer stage* early diagno?s" or "earlier diagno?s")	52,030
S10	S8 OR S9	59,476
S11	(MH "Diagnosis, Delayed")	6,164
S12	(MH "Early Detection of Cancer/ED/EV/MT/MO/NU/OG/PF/ST/SN/TD/UT")	5,142
S13	(MH "Early Diagnosis+")	33,337
S14	(MH "Neoplasm Grading") OR (MH "Neoplasm Staging") OR (MH "Nursing Assessment") OR (MH "Nursing Diagnosis")	68,343
S15	(MH "Early Detection of Cancer")	14,436

S16	(MH "Early Diagnosis+")	33,337
S17	(MH "Diagnosis, Delayed")	6,164
S18	TI ("early detection" or "early diagnosis" or "prediagnosis" or "prediagnosis" or "early referral*" or "earlier referral*" or "rapid referral*" or "fast referral*" or "fast track referral*" or "fast referral* referral route" or "fast track" or "referral path?" or "referral interval" or "cancer pathway*" or "cancer care pathway*" or "cancer path?" or "timely detection" or "early identification" or "timeliness" or "time-to-treat*" or "patient interval" or "patient care interval" or "priamary care interval" or "general practitioner interval" or "GP interval" or "consult* interval" or "diagnostic* interval" or "time to diagnose*" or "diagnose* stage" or "cancer stage* early diagno?s" or "earlier diagno?s")	10,693
S19	AB ("early detection" or "early diagnosis" or "prediagnosis" or "prediagnosis" or "early referral*" or "earlier referral*" or "rapid referral*" or "fast referral*" or "fast track referral*" or "fast referral* referral route" or "fast track" or "referral path?" or "referral interval" or "cancer pathway*" or "cancer care pathway*" or "cancer path?" or "timely detection" or "early identification" or "timeliness" or "time-to-treat*" or "patient interval" or "patient care interval" or "priamary care interval" or "general practitioner interval" or "GP interval" or "consult* interval" or "diagnostic* interval" or "time to diagnose*" or "diagnose* stage" or "cancer stage* early diagno?s" or "earlier diagno?s")	52,030
S20	S15 OR S16 OR S17 OR S18 OR S19	87,947
S21	(MH "Early Intervention+")	24,456
S22	(MH "Nursing Interventions")	12,065
S23	psychosocial intervention	6,771
S24	(MH "Psychosocial Intervention")	2,724
S25	(MH "Internet-Based Intervention")	2,521
S26	S21 OR S22 OR S23 OR S24 OR S25	45,388
S27	TI (intervention* or initiativ* or strateg* or campaign* or aware* or programs* or train*)	388,825
S28	AB (intervention* or initiativ* or strateg* or campaign* or aware* or programs* or train*)	1,222,973
S29	S27 OR S28	1,418,068
S30	S26 OR S29	1,436,336
S31	(MH "Lung Neoplasms+")	56,256

S32	TI (((lung or pulmonary or bronch*) N2 (cancer* or neopla* or carcino* or malig* or metasta*))) OR AB (((lung or pulmonary or bronch*) N2 (cancer* or neopla* or carcino* or malig* or metasta*)))	70,223
S33	S31 OR S32	86,774
S34	S20 AND S30 AND S33	1,039
S35	TI Pathways to lung cancer diagnosis: a qualitative study of patients and general practitioners about diagnostic and pretreatment intervals	1
S36	S34 AND S35	1
S37	(MH "Stomach Neoplasms")	13,066
S38	TI (((gastr* or gut or stomach*) N2 (carcin* or cancer* or neoplas* or tumo* or growth* or adenocarcin* or malig*))) OR AB (((gastr* or gut or stomach*) N2 (carcin* or cancer* or neoplas* or tumo* or growth* or adenocarcin* or malig*)))	25,134
S39	S37 OR S38	27,796
S40	S20 AND S30 AND S39	278
S41	(MH "Pancreatic Neoplasms+")	14,911
S42	TI ((pancreas* N2 (neoplas* or cancer* or carcin* or tumo* or metasta* or malig*))) OR AB ((pancreas* N2 (neoplas* or cancer* or carcin* or tumo* or metasta* or malig*)))	1,684
S43	S41 OR S42	15,532
S44	S20 AND S30 AND S43	223
S45	(MH "Ovarian Neoplasms+")	16,388
S46	TI (((ovarian* or ovary*) N2 (neoplas* or cancer* or carcin* or tumo* or metasta* or malig*))) OR AB (((ovarian* or ovary*) N2 (neoplas* or cancer* or carcin* or tumo* or metasta* or malig*)))	18,520
S47	S45 OR S46	22,983
S48	S20 AND S30 AND S47	314
S49	S34 OR S40 OR S44 OR S48	1,759
S52	S34 OR S40 OR S44 OR S48 AND (YR 2024-2026)	1,532
